# Changes in Corneal Volume at Different Areas and Its Correlation with Corneal Biomechanics after SMILE and FS-LASIK Surgery

**DOI:** 10.1155/2020/1713979

**Published:** 2020-04-27

**Authors:** Pinghui Wei, George PM Cheng, Jiamei Zhang, Alex LK Ng, Tommy CY Chan, Vishal Jhanji, Yan Wang

**Affiliations:** ^1^Clinical College of Ophthalmology, Tianjin Medical University, Tianjin, China; ^2^Tianjin Eye Hospital, Tianjin Eye Institute, Tianjin Key Lab of Ophthalmology and Visual Science, Nankai University, Tianjin, China; ^3^Hong Kong Laser Eye Center, Hong Kong, China; ^4^Department of Ophthalmology, The University of Hong Kong, Hong Kong, China; ^5^Department of Ophthalmology and Visual Sciences, The Chinese University of Hong Kong, Hong Kong, China; ^6^UPMC Eye Center, University of Pittsburgh School of Medicine, Pittsburgh, PA, USA

## Abstract

**Purpose:**

To investigate the variations of corneal volume (CV) after small incision lenticule extraction (SMILE) and femtosecond laser-assisted laser in situ keratomileusis (FS-LASIK) and analyze the influences of biomechanical properties on the changes of refraction and CV.

**Methods:**

Ninety-seven eyes of 97 patients undergoing SMILE and FS-LASIK were included in this retrospective study. CV was measured with Scheimpflug-based corneal topography at preoperatively and at day 1, week 1, and months 1 and 3 postoperatively. CV measured within 5 mm diameter was defined as central region volume (CV5) and between 5 mm and 10 mm diameter was defined as peripheral region volume (CV5-10). An Ocular Response Analyzer (ORA) was used to assess corneal biomechanical properties including corneal hysteresis (CH) and corneal resistant factor (CRF). The reduction of study parameters (△) were calculated by subtracting the preoperative value at various time points from the postoperative values.

**Results:**

CV had significant reduction after the SMILE and FS-LASIK procedure (*P* < 0.05). CV5 increased significantly from postoperative day 1 to month 3 (*P* < 0.001) in SMILE, while both CV5 and CV5-10 increased significantly in FS-LASIK (*P* < 0.001). The increase in CV5 after SMILE was 0.11 ± 0.16 mm^3^，which was significantly different from FS-LASIK (0.20 ± 0.13 mm^3^, *P*=0.004). In the SMILE group, △CV5 correlated with △CRF (*r* = 0.498, *P* < 0.001) and △CH (*r* = 0.374, *P*=0.007). In the FS-LASIK group, △CV5 and △CRF had a significant correlation (*r* = 0.363, *P*=0.012), but not with △CH.

**Conclusions:**

Dynamic changes in corneal volume were found after SMILE and FS-LASIK surgery. The central region significantly increased after SMILE, while both central and peripheral regions increased following FS-LASIK in the early postoperative period. SMILE was associated with less change in biomechanical properties per unit of reduction in CV compared with FS-LASIK.

## 1. Introduction

Corneal volume (CV) is one of the structural characteristics contributing to the biomechanical profile of the cornea [1]. Corneal refractive surgery involves ablation of the corneal tissue thereby leading to a reduction in the corneal volume [2]. Several published studies use corneal thickness to evaluate the amount of tissue changed after refractive surgery and to study its relationship with refractive outcome [2, 3]. However, a change in corneal thickness does not truly reflect the actual change in the amount of corneal tissues. On the other hand, evaluation of changes in CV may be a more comprehensive approach to study the actual amount of changes in the corneal tissue as a whole and characterize corneal morphometric changes with a single value [4].

Currently, the main applications of femtosecond laser in corneal refractive surgery include femtosecond laser-assisted laser in situ keratomileusis (FS-LASIK) and small incision lenticule extraction (SMILE). FS-LASIK creates a corneal flap with femtosecond laser followed by excimer laser ablation of the stromal tissue, while a stromal lenticule is created with femtosecond laser and then removed through a small incision in SMILE. Corneal tissue is removed and CV is reduced in both methods, which is followed by change in the corneal shape and correction of refractive errors; hence, corneal ectasia might occur [5–7]. Studies have shown a correlation between corneal volume and corneal biomechanical properties [8, 9]. However, there is still little understanding regarding the dynamic changes in the CV after SMILE and FS-LASIK [10].

To the best of our knowledge, there is no study focusing on the changes in CV at different areas of the cornea and analyzing its correlation with corneal biomechanical properties after SMILE and FS-LASIK.

## 2. Methods

### 2.1. Subjects and Examinations

This was a retrospective, comparative study that included patients with myopia and myopic astigmatism undergoing FS-LASIK and SMILE in Tianjin Eye Hospital. Inclusion criteria included age of 18 years or older, a corrected distance visual acuity (CDVA) of 20/25 or better, myopic spherical refraction from −0.50 to −10.00 diopters (D), myopic cylindrical refraction up to −3.00 *D*, stable refraction over 2 years, and central corneal thickness more than 480 *μ*m. All patients had stopped soft contact lens wear for at least 2 weeks and rigid lens for at least 4 weeks before the assessment. Exclusion criteria included active ocular disease, history of ocular surgery or trauma, keratoconus or suspicious corneal topography, and patients with mental disorders. The study protocol was approved by the Tianjin Eye Hospital Ethics Committee and adhered to the Declaration of Helsinki. Written informed consent was obtained from all patients before the surgery.

### 2.2. Measurement Methods

Clinical examinations were performed preoperatively and on postoperative day 1, week 1, month 1, and month 3. Preoperative and postoperative examinations included measurement of uncorrected visual acuity (UCVA), CDVA, eye dominance, noncontact tonometry, slit lamp biomicroscopy examination, and dilated fundus examination. All patients underwent CV measurement by Pentacam HR topography (Oculus GmbH, Wetzlar, Germany). The Pentacam was performed in a dark room, and only the scans with quality specification screen displaying “OK” were chosen for analysis. The changes in the CV before and after surgery were calculated. An ocular response analyzer (ORA, Reichert, USA) was used to measure corneal biomechanical properties including corneal hysteresis (CH) and corneal resistance factor (CRF). Measurements below a waveform score of five were excluded due to insufficient quality.

### 2.3. Surgical Technique

All surgeries were performed by the same surgeon (WY) using a 500 kHz femtosecond laser machine (Visumax, Carl Zeiss Meditec AG, Germany). In FS-LASIK, the flap thickness was 110 *μ*m and the flap diameter was 7.9–8.0 mm. The excimer laser ablation was performed with an Allegretto (Wavelight Laser Technologie AG, Germany). In SMILE, the lenticule diameter was 6.5 mm, the cap thickness was 110 *μ*m, and the incision location was at the 12 o'clock position. After the surgery, 0.3% ofloxacin (Tarivid; Santen, Inc, Japan) eye drops were administrated four times a day for three days and 0.1% fluorometholone (Flumetholon; Santen, Inc, Japan) eye drops were given four times per day for the first two weeks and then decreased one time every two weeks.

### 2.4. Statistical Analysis

Statistical analysis was performed using SPSS (GLM UNIVARIATE, version 20, IBM). All data were tested with the Kolmogorov–Smirnov test and were normally distributed. We divided the cornea into 2 regions: central (CV5, the central 5 mm diameter region) and peripheral (CV5-10, the 5 mm to 10 mm diameter region). The reduction in the study parameters (△) was calculated by subtracting the preoperative value at various time points from the postoperative values. The differences in the corneal volume between the SMILE and FS-LASIK group were calculated using the independent *t*-test; the Pearson correlation test was used to evaluate the relationship between the change in corneal volume (△CV) and the change in spherical equivalence refraction (△SE), change in corneal hysteresis (△CH), and change in corneal resistance factor (△CRF). One-way repeated measures analysis of variance (ANOVA) with the post hoc Bonferroni test was applied for multiple comparisons between different time points. *P* < 0.05 was regarded as statistically significant.

## 3. Results

This study included 97 eyes (97 patients; 50 eyes in SMILE and 47 eyes in FS-LASIK). The average age was 24.28 ± 5.86 (range 18–41). The preoperative characteristics are shown in Table 1.

### 3.1. CV Changes on Postoperative Day 1, Week 1, Month 1, and Month 3

As shown in Table 2, there was a statistically significant reduction in CV5 and CV5-10 on postoperative day 1 compared with the preoperative value (all *P* < 0.05) in both groups. In SMILE, CV5-10 continued to decline at postoperative week 1 (*P*=0.039), while a significant decrease in CV5 was observed in FS-LASIK (*P*=0.024), when compared with postoperative day 1. Afterwards, there was a gradual increase of both CV5 and CV5-10 from postoperative week 1 until the end of 3 months.

### 3.2. Comparison of the Corneal Volume between the SMILE and FS-LASIK Group

CV5 increased significantly from postoperative day 1 to month 3 (*P* < 0.001) in SMILE, while both CV5 and CV5-10 increased significantly in FS-LASIK (*P* < 0.001). The increase in CV5 from postoperative day 1 to month 3 in the SMILE group was 0.11 ± 0.16 mm^3^，which was significantly different from that in the FS-LASIK group (0.20 ± 0.13 mm^3^, *t* = −2.917, *P*=0.004). The corresponding increase in the CV5-10 in SMILE and FS-LASIK group was 0.26 ± 0.90 mm^3^ and 0.54 ± 0.77 mm^3^, respectively (*t* = −1.599, *P*=0.113).

### 3.3. Changes in Spherical Equivalent Refraction at Postoperative Day 1, Week 1, Month 1, and Month 3

**Figure 1:**
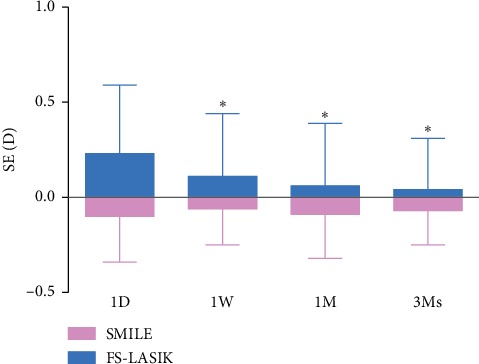
The postoperative spherical equivalence refraction (SE) at different time points. Asterisk (^*∗*^): significant difference was found when compared with the postoperative day 1 value in the FS-LASIK group (post hoc Bonferroni test, (*P* < 0.05)).

The preoperative spherical equivalence refraction (SE) in the SMILE and FS-LASIK group was -5.90 ± 1.33D and −6.15 ± 1.72D, respectively. The change in spherical equivalence refraction at postoperative month 3 (△SE) was −5.82 ± 1.29D and −6.19 ± 1.76D, respectively, and no statistically significant difference was found between the two groups (*t* = 1.153, *P*=0.252). The SE at postoperative day 1, week 1, month 1, and months 3 in SMILE was -0.10 ± 0.24D, −0.05 ± 0.19D, −0.09 ± 0.23D, and −0.07 ± 0.18D, respectively，and no statistically significant difference was found between these values (*P* > 0.05). The corresponding values in FS-LASIK were 0.23 ± 0.36D, 0.11 ± 0.33D, 0.06 ± 0.33D, and 0.04 ± 0.27D. There was a statistically significant difference between the SE at postoperative day 1 and all other visits (*P* < 0.05) (Figure 1).

### 3.4. Correlation between Change in Spherical Equivalence Refraction and Reduction in Corneal Volume

**Figure 2:**
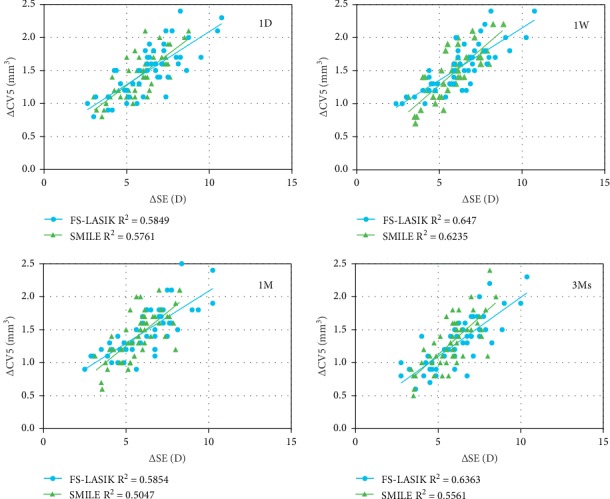
The correlation between the change in volume of the central 5 mm diameter area (△CV5) and the change in the spherical equivalence refraction (△SE) at different time points.

A significant correlation was found between the change in CV5 at postoperative month 3 compared with preoperative value, and the change of SE at postoperative month 3 compared with the preoperative value (SMILE group: *r* = 0.746, *P* < 0.001; FS-LASIK group: *r* = 0.798, *P* < 0.001) (Figure 2). No correlation was found between the change in CV5 from postoperative day 1 and month 3 and change in the corresponding SE (SMILE group: *r* = 0.044, *P*=0.760; FS-LASIK group: *r* = 0.114, *P*=0.447).

### 3.5. Correlation between Change in Corneal Biomechanical Parameters and Reduction in Corneal Volume

The mean reduction of CRF and CH (△CRF and △CH) in the SMILE group was 3.58 ± 1.02 mmHg and 2.12 ± 1.00 mmHg, while in the FS-LASIK group, the corresponding values were 3.96 ± 1.07 mmHg and 2.60 ± 1.00 mmHg, respectively. As shown in Table 3, in the SMILE group, a statistically significant correlation was found between △CV5 with △CRF (*r* = 0.498, *P* < 0.001) and △CH (*r* = 0.374, *P* < 0.001) at 3 months. In the FS-LASIK group, only △CV5 and △CRF had a statistically significant correlation (*r* = 0.363, *P*=0.012).

### 3.6. Correlation between the Change in Corneal Biomechanical Parameters and Change in Spherical Equivalent

As shown in Table 3, a statistically significant correlation was found between △SE with △CRF at 3 months postoperatively.

## 4. Discussion

Corneal volume is an important quantitative parameter for monitoring the change in the corneal tissue characteristics after surgery [11]. Precise knowledge about the actual amount of tissue ablated during surgery may help understand the predictability of the surgery. Argentoet al. [12] and Pallikaris et al. [13] reported that the amount of tissue removed during surgery which is the same as the reduction in the CV could be a predicting factor for the development of corneal ectasia. CV has been used together with other parameters to improve the sensitivity and specificity for diagnosis of keratoconus [4–7]. Gatinel et al. [14] used a geometrical model to estimate the change in CV after corneal refractive surgery and found a relationship between the change in CV with the size of optical zone and the magnitude of refractive error to be corrected. The Pentacam uses a rotating Scheimpflug camera to reconstruct the 3D image of the anterior segment and can be used to obtain corneal thickness and CV data with good repeatability and consistency [4]. The corneal thickness measurement of the Pentacam is comparable to ultrasound pachymetry [15], with good accuracy [16, 17] and high repeatability [18]. To the best of our knowledge, there are no studies that utilized Pentacam to compare the changes in CV after SMILE and FS-LASIK. CV might be more sensitive to reflect corneal profile changes than corneal thickness, since inflammatory response and corneal wound healing response after surgery would not be localized.

Our study found that for both procedures, CV changes with time. The CV5 and CV5-10 decreased in postoperative day 1 and continued to decrease at week 1, followed by a gradual increase at month1 and month 3. From day 1 to week 1, the CV decreased in the peripheral region but not in centrally after SMILE, while following FS-LASIK, it decreased centrally not peripherally. The negative pressure suction, irrigation, and manipulation on the corneal stroma may all lead to corneal edema on postoperative day 1. Studies have reported that the central corneal haze early after SMILE could be due to edema in the corneal stromal interface [19]. Since the flap in FS-LASIK is no longer in tension, it was more difficult to recover due to changes in the swelling pressure associated with loss of tension. In SMILE, the cap can maintain some tension after surgery and would thus be able to recover more quickly [20]. The edema usually subsides in 1 week, which explains the slight reduction in corneal volume. Afterwards, the healing response and inflammatory response in the cornea will lead to proliferation of corneal stromal collagen fibers, and this could account for the increase in CV at month 1.

The increase in CV at postoperative month 3 compared with day 1 was larger in the FS-LASIK group compared with the SMILE group. SMILE had an increase in CV centrally, while FS-LASIK showed the increase both centrally and peripherally. Both the changes of epithelial and stroma thickness may contribute to the increment of CV. Previous studies [21, 22] have shown that the epithelial thickness (ET) at postoperative day 1 and month 3 in SMILE was 53.6 ± 3.3 *μ*m and 58.0 ± 3.7 *μ*m, respectively; while in the FS-LASIK group, the corresponding value was 52.43 ± 3.1 *μ*m and 56.42 ± 5.6 *μ*m, respectively. The increase in ET from postoperative day 1 to month 3 in the SMILE group and FS-LASIK group was about 4.4 *μ*m and 4 *μ*m, respectively. Such little variations seem unable to contribute to the significant differences of CV between two groups. We speculated that this difference might be related to the different corneal wound healing responses in both surgeries. A previous animal eye study has shown that when compared with LASIK, the refractive lenticule extraction (ReLEx) procedure may result in less inflammation and early extracellular matrix deposition [23]. Another study that compared early corneal wound healing and inflammatory responses between FS-LASIK and SMILE has shown that, in SMILE, there were significantly fewer Ki67-positive cells and CD11b-positive cells [24]. They also reported that SMILE induced less keratocyte apoptosis than LASIK.

**Figure 3:**
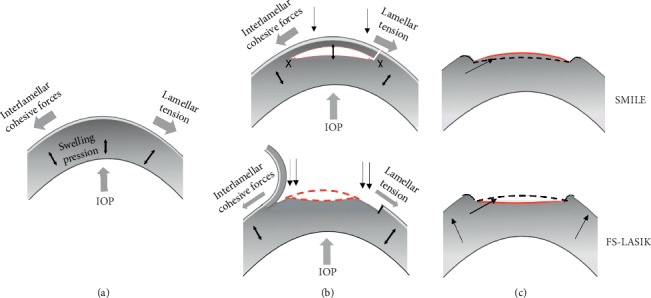
Schematic diagrams showing the effect of corneal biomechanical properties on the postoperative spherical equivalent refraction. (a) Before operation，there was a balance between the corneal swelling pressure with the interlamellar cohesive force and the lamellar tension; (b) intraoperatively, only side cuts around the lenticule were made in SMILE; in FS-LASIK, the central anterior stroma was cut, and x indicates where the collagen was cut; and (c) The black line included the simulated corneal curvature postoperatively, and the red line included the actual corneal curvature.

We analyzed the changes in CV at different time points and their relation with SE. In SMILE, there was less change in the SE and no statistical differences existed between the SE at each time points. In the FS-LASIK group, there was mild overcorrection at postoperative day 1. At subsequent visits, the SE showed myopic shift, and this was demonstrated by a coincidence with an increase in the corneal volume. In other words, the increase in CV at postoperative month 3 is correlated with the corresponding myopic shift. The early overcorrection and subsequent myopic shift in FS-LASIK could also be related to the postoperative changes in the corneal biomechanical properties. Our study found a close relationship between the change in CV and the biomechanical properties, and this change in the corneal structure and shape will eventually lead to change in the refraction [25]. The cornea is a heterogeneous viscoelastic biological material. Under normal circumstances, the interlamellar cohesive force, the lamellar tension, and the intraocular pressure balance out the corneal swelling pressure and maintain the biomechanical stability (Figure 3(a)). A reduction in corneal biomechanical properties has been shown after SMILE and FS-LASIK [26], but this effect was less in SMILE than in LASIK [2, 26]. We also demonstrated less change in CH and CRF for a similar change in SE in SMILE compared to FS-LASIK in the current study. We hypothesize that, in SMILE, only the collagen fibers at the side-cut region around the lenticule were cut, whereas the collagen fibers in the anterior stroma remained intact. The interlamellar cohesive force and lamellar tension in the corneal stromal interface were reduced after lenticule extraction, which decrease local resistance to corneal swelling in the interface and result in stromal thickening between the anterior cap and the residual stroma. This led to a slightly less central flattening compared to what is predicted (Figures 3(b) and 3(c)). As for FS-LASIK, the collagen fibers in the anterior stroma were cut and lamellar layers within the flap can no longer bear tension which would cause a greater change in the corneal biomechanical properties [19]. Cutting the central portion of the cornea released the lamellar tension in the peripheral corneal stroma, leading to an increase in the peripheral corneal thickness and increased the cohesive pulling force from the periphery to the central stromal region. This likely led to excess central flattening biomechanically than expected and, therefore, overcorrection early after FS-LASIK. This was also shown in our study that only central region significantly increased from postoperative day 1 to month 3 after SMILE, while both central and peripheral regions increased following FS-LASIK. Avunduk et al. [27] reported that after refractive surgery, the healing response at the keratocyte activation zone, the rearrangements of collagen fibers during corneal remodeling, and the corneal biomechanical changes may all cause changes in refraction as well as the anterior and posterior corneal curvature.

We reported that at postoperative month 3, the reduction in corneal volume at different regions in SMILE correlated with the △CRF and △CH, whereas in FS-LASIK, only the △CV5 correlated with the △CRF, and no correlation was found between the corneal volume change in other regions and the reduction in corneal biomechanical properties. This is mainly due to the reduction in the amount of corneal collagen fibers and reduction of extracellular matrix components consequent to the reduction in corneal volume. Also, from Figure 2, we could see that SMILE had less change in biomechanical properties per unit of reduction in CV when compared with FS-LASIK. Apart from this, in both SMILE and FS-LASIK, the reduction in corneal volume had a stronger correlation with △CRF than with △CH. Chen et al. reported that after LASIK, the ablation depth correlated with the CRF parameter but not with the CH parameter, and pointed that CRF may be more useful than CH in assessing the biomechanical changes after LASIK [28].

In conclusion, there were dynamic changes in the corneal volume after SMILE and FS-LASIK during the early postoperative period. From day 1 to week 1, the CV decreased in the peripheral region but not centrally after SMILE, while following FS-LASIK, it decreased centrally not peripherally. The SMILE group had an increase in CV centrally from postoperative day 1 to month 3, while the FS-LASIK group showed the increase both centrally and peripherally. SMILE was associated with less change in biomechanical properties per unit of reduction in CV when compared with FS-LASIK. These postoperative corneal volume and biomechanics changes could be associated with the changes in the spherical equivalent. Further studies are warranted in future to confirm our findings.

## Figures and Tables

**Table 1:** Baseline characteristics of eyes undergoing SMILE and FS-LASIK.

Parameters	SMILE	FS-LASIK	t	*P* value
MRSE (D)	−5.90 ± 1.33	−6.15 ± 1.72	0.804	0.423
Spherical (D)	−5.53 ± 1.36	−5.78 ± 1.69	0.814	0.418
Cylinder (D)	−0.86 ± 0.74	−0.76 ± 0.59	−0.657	0.513
Age (y)	24.56 ± 6.02	23.97 ± 5.95	0.486	0.628
CCT (*μ*m)	545.70 ± 29.70	540.97 ± 25.21	0.841	0.402

MRSE: manifest refraction spherical equivalent, CCT: central corneal thickness.

**Table 2:** Corneal volume during postoperative visits in patients undergoing SMILE and FS-LASIK.

Parameters	Group	Preop	1 Day	1 Week	1 Month	3 Months	*P* value
CV5 (mm^3^)	SMILE	11.56 ± 0.64	10.13 ± 0.63^*∗*^	10.09 ± 0.65^*∗*^	10.14 ± 0.65^*∗*^	10.25 ± 0.66^*∗#*^	<0.001
	FS-LASIK	11.47 ± 0.52	9.96 ± 0.51^*∗*^	9.91 ± 0.50^*∗#*^	9.98 ± 0.50^*∗*^	10.16 ± 0.49^*∗#*^	<0.001
CV5-10 (mm^3^)	SMILE	49.69 ± 2.78	48.89 ± 2.79^*∗*^	48.60 ± 2.68^*∗#*^	48.67 ± 2.76^*∗*^	49.15 ± 2.80^*∗*^	<0.001
	FA-LASIK	49.17 ± 2.27	48.28 ± 2.41^*∗*^	48.07 ± 2.39^*∗*^	48.10 ± 2.33^*∗*^	48.81 ± 2.41^#^	<0.001

CV5: corneal volume of the central 5 mm diameter area, CV5-10: corneal volume of the peripheral 5–10 mm diameter area, *P* value: one-way repeated measures analysis of variance (ANOVA), ^*∗*^Significant difference when compared with preoperative value (*P* < 0.05), ^#^ Significant difference when compared with postoperative day 1 (*P* < 0.05).

**Table 3:** Correlation between the reduction of corneal volume and spherical equivalent with the change in corneal hysteresis and corneal resistance factor at 3 months.

Parameters	SMILE		FS-LASIK	
	r	*P* value	r	*P* value
△CV5 VS △CRF	0.498^*∗*^	<0.001	0.363^*∗*^	0.012
△CV5-10 VS△CRF	0.270	0.058	0.125	0.403
△CV5 VS △CH	0.374^*∗*^	0.007	0.264	0.073
△CV5-10 VS △CH	0.116	0.420	0.098	0.512
△CV5 VS △SE	−0.746^*∗*^	<0.001	^−^0.798^*∗*^	<0.001
△CV5-10 VS △SE	^−^0.353^*∗*^	0.012	^−^0.440^*∗*^	0.002
△SE VS △CRF	^−^0.559^*∗*^	<0.001	^−^0.598^*∗*^	<0.001
△SE VS △CH	^−^0.506^*∗*^	<0.001	^−^0.472^*∗*^	0.001

CRF: corneal resistance factor，CH: corneal hysteresis, CV5: corneal volume of the central 5 mm diameter area, CV5-10: corneal volume of the peripheral 5–10 mm diameter area, SE: spherical equivalent, △: the reduction at postoperative month 3, ^*∗*^*P* < 0.05(Pearson correlation).

## Data Availability

The data that support the findings of this study are available from the corresponding author upon reasonable request.
